# Correction: De Jesus et al. Body Temperature Drop as a Humane Endpoint in Snake Venom-Lethality Neutralization Tests. *Toxins* 2023, *15*, 525

**DOI:** 10.3390/toxins16010025

**Published:** 2024-01-03

**Authors:** Rosa De Jesus, Adam E. Tratner, Alanna Madrid, Andrés Rivera-Mondragón, Goy E. Navas, Ricardo Lleonart, Gabrielle B. Britton, Patricia L. Fernández

**Affiliations:** 1 Bioterio, Instituto de Investigaciones Científicas y Servicios de Alta Tecnología (INDICASAT AIP), City of Knowledge, Panama City 0843-01103, Panama; rdejesus@indicasat.org.pa (R.D.J.); amadrid@indicasat.org.pa (A.M.); 2 Florida State University, Republic of Panama Campus, City of Knowledge, Panama City 0843-01103, Panama; atratner@fsu.edu; 3 Centro de Neurociencias, INDICASAT AIP, City of Knowledge, Panama City 0843-01103, Panama; 4 Instituto Especializado de Análisis (IEA), Universidad de Panamá, Panama City P.O. Box 3366, Panama; andres.riveraa@up.ac.pa (A.R.-M.); goy.navast@up.ac.pa (G.E.N.); 5 Centro de Biología Celular y Molecular de Enfermedades, INDICASAT AIP, City of Knowledge, Panama City 0843-01103, Panama; rlleonart@indicasat.org.pa

## Error in Figure

In the original publication [1] there was a mistake in Figure 1. The Kaplan–Meier survival curve was constructed by assessing the mortality of the animals at 1, 2, 3, 24, and 48 h. However, the plotting software applied a default configuration associated with the kind of graph used by which the x axis scale was changed to represent a regular time interval of 1 h (values: 0, 1, 2, 3, 4, and 5). The original figure is as follows:




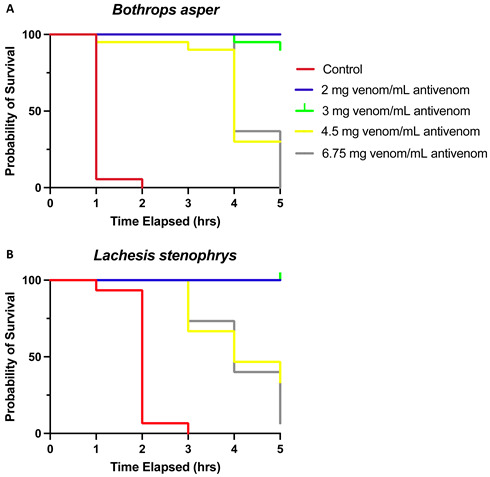


**Figure 1.** Kaplan–Meier survival analysis of (**A**) *B. asper* and (**B**) *L. stenophrys* venoms stratified into five groups (controls and 2, 3, 4.5, and 6.75 mg venom/mL antivenom) shows a significantly better overall survival for groups receiving lower venom/antivenom dosages. Lethality rates were not different between venoms.



Nevertheless, the correct values are: 0, 1, 2, 3, 24, and 48 h. The corrected Figure 1 is presented below. The authors affirm that the scientific conclusions remain unchanged. The original publication has been updated.

## Figures and Tables

**Figure 1:**
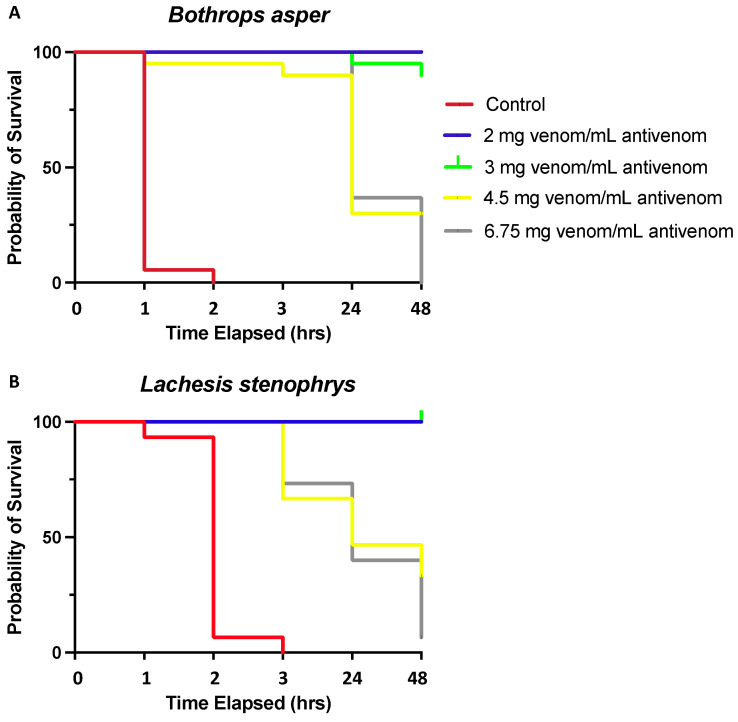
Kaplan–Meier survival analysis of (**A**) *B. asper* and (**B**) *L. stenophrys* venoms stratified into five groups (controls and 2, 3, 4.5, and 6.75 mg venom/mL antivenom) shows a significantly better overall survival for groups receiving lower venom/antivenom dosages. Lethality rates were not different between venoms.
