# Supplementary material for: Body Temperature Drop as a Humane Endpoint in Snake Venom-Lethality Neutralization Tests
Source: Toxins (Basel). 2023 Aug 26;15(9):525. doi: 10.3390/toxins15090525 (PMC10535418; doi:10.3390/toxins15090525)
Supplement: Supplementary file 1 [file toxins-15-00525-s001.zip › toxins-2499115-supplementary.pdf]

Supplementary material

Title: **Body temperature drop as a humane endpoint in antivenom lethality neutralization tests.**

Authors: **Rosa De Jesus†, Adam E. Tratner†, Alanna Madrid, Andrés Rivera, Goy E. Navas, Ricardo Lleonart, Gabrielle B. Britton*, Patricia L. Fernández***

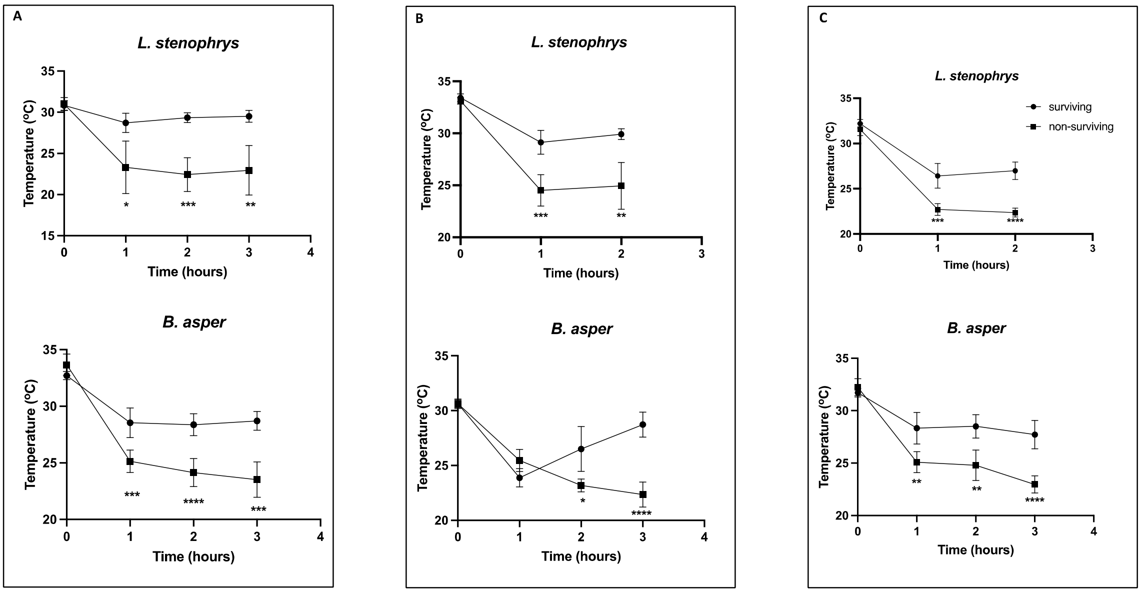

Figure S1: Temperature differences between surviving and non-surviving mice for experiment and each venom type. Groups of 5 mice were inoculated with mixtures of a fixed dose of venom (4xLD50) and variable doses of antivenom (2, 3, 4.5 and 6.75 mg venom/mL antivenom). Body temperature was recorded before inoculation and every hour for 3 hours. Graphs represent mean ± 95% CI of temperature from surviving and non-surviving mice for independent experiments (A) experiment 1, (B) experiment 2 and (C) experiment 3.

Table S1: Cumulative mortality and body temperature by venom type, group (dosage) and time.

			Temperature M/SD and Mortality (n, % mortality)					
	n Deceased	*n* Survived	Baseline	1 hr	2 hr	3 hr	24 hr	48 hr
Bothrops asper	50	43						
2 mg venom/mL antivenom	1	19	31.53/.99 (0, 0%)	27.53/3.19 (1, 5.0%)	28.77/1.77 (1, 5.0%)	29.39/1.01 (1, 5.0%)	30.67/1.50 (1, 5.0%)	31.98/1.34 (1, 5.0%)

3 mg venom/mL antivenom	2	18	31.67/1.01 (0, 0%)	26.88/2.10 (0, 0%)	27.21/2.30 (0, 0%)	26.81/2.26 (1, 5.0%)	28.99/3.50 (1, 5.0%)	31.38/2.23 (2, 10.0%)
4.5 mg venom/mL antivenom	13	6	31.73/.93 (0, 0%)	24.95/1.36 (0, 0%)	24.95/2.19 (0, 0%)	24.67/3.01 (5, 26.3%)	24.24/3.63 (8, 42.1%)	29.03/3.52 (13, 68.4%)
6.75 mg venom/mL antivenom	20	0	32.2/1.66 (0, 0%)	24.88/2.26 (0, 0%)	23.26/2.24 (0, 0%)	23.13/1.65 (5, 25.0%)	23.50/3.27 (12, 6.0%)	-- (20, 100%)
Control (venom only)	14	0	31.91/1.24 (0, 0%)	21.00/.00 (13, 92.9%)	-- (14, 100%)	-- (14, 100%)	-- (14, 100%)	-- (14, 100%)
Lachesis stenophrys	40	34						
2 mg venom/mL antivenom	1	14	32.44/1.38 (0, 0%)	29.45/1.25 (0, 0%)	29.29/1.65 (0, 0%)	30.68/1.30 (0, 0%)	31.51/1.50 (0, 0%)	31.68/.71 (1, 6.7%)
3 mg venom/mL antivenom	0	15	32.10/1.32 (0, 0%)	26.81/2.17 (0, 0%)	28.37/1.75 (0, 0%)	29.02/.52 (0, 0%)	30.17/1.99 (0, 0%)	31.06/2.34 (0, 0%)
4.5 mg venom/mL antivenom	10	5	31.72/1.21 (0, 0%)	26.18/3.31 (0, 0%)	26.24/3.11 (0, 0%)	28.60/.66 (0, 0%)	27.87/4.81 (8, 53.3%)	31.62/.89 (10, 66.7%)
6.75 mg venom/mL antivenom	14	0	32.35/1.11 (0, 0%)	22.71/1.33 (0, 0%)	22.41/1.18 (0, 0%)	21.82/1.00 (9, 64.29%)	21.18/.85 (9, 64.3%)	-- (14, 100%)
Control (venom only)	15	0	32.01/.79 (0, 0%)	21.74/.71 (1, 6.7%)	20.30/.00 (14, 93.3%)	-- (15, 100%)	-- (15, 100%)	-- (15, 100%)
Total	90	77	31.95/1.19 (0, 0%)	25.74/3.03 (15, 9.0%)	26.25/3.16 (29, 17.4%)	26.37/3.30 (50, 29.9%)	28.43/4.09 (68, 40.7%)	31.35/2.02 (90, 53.9%)

Table S2: Hypothetical ED_{50} values at different time points for each experiment.

		ED_{50} (CI 95%)*		
Venom	Time (h)	Exp. 1	Exp. 2	Exp. 3
Bothrops asper	1	4.32 (3.63 - 5.14)	N/A	3.8 (2.53 - 5.68)
	2	3.98 (3.11 - 5.09)	2.8 (2.00 - 3.92)	3.98 (3.18 - 4.98)
	3	5.08 (4.37 - 5.9)	3.21 (2.54 - 4.06)	3.12 (2.62 - 3.71)
Lachesis stenophrys	1	5.08 (4.40 - 5.85)	3.38 (2.70 - 4.24)	2.44
	2	5.51	4.68 (3.83 - 5.72)	3.25 (2.45 - 4.31)
	3	5.51	N/A	N/A

* mg venom neutralized/mL antivenom

Table S3: ED_{50} values for independent experiments.

Venom	ED_{50} (CI 95%)*		
	Exp. 1	Exp. 2	Exp. 3
Bothrops asper	5.62	3.61 (2.52-5.19)	3.75
Lachesis stenophrys	5.08 (4.41-5.86)	3.98 (3.45-4.59)	3.38 (2.93-3.91)

* mg venom neutralized/mL antivenom
